# Correlation between matrix structural order and compressive stress exerted on silicon nanocrystals embedded in silicon-rich silicon oxide

**DOI:** 10.1186/1556-276X-8-40

**Published:** 2013-01-21

**Authors:** Grzegorz Zatryb, Artur Podhorodecki, Jan Misiewicz, Julien Cardin, Fabrice Gourbilleau

**Affiliations:** 1 Institute of Physics, Wroclaw University of Technology, Wybrzeze Wyspianskiego 27, 50-370, Wroclaw, Poland; 2 CIMAP, UMR CNRS/CEA/ENSICAEN/UCBN, Ensicaen 6 Blvd Maréchal Juin, 14050, Caen Cedex 4, France

**Keywords:** Silicon, Nanocrystals, Stress, Raman, Phonon, Confinement, Order, Disorder, Matrix

## Abstract

**Abstract:**

Silicon nanocrystals embedded in a silicon oxide matrix were deposited by radio frequency reactive magnetron sputtering. By means of Raman spectroscopy, we have found that a compressive stress is exerted on the silicon nanocrystal cores. The stress varies as a function of silicon concentration in the silicon-rich silicon oxide layers varies, which can be attributed to changes of nanocrystal environment. By conducting the Fourier transform infrared absorption experiments, we have correlated the stresses exerted on the nanocrystal core to the degree of matrix structural order.

**PACS:**

78.67.Bf, 78.67.Pt, 73.63.Bd, 78.47.D, 74.25.Nd

## Background

Silicon nanocrystals (Si-NCs) embedded in a silicon-rich silicon oxide (SRSO) have been extensively studied due to their promising applications in the third generation tandem solar cells [1], light-emitting diodes [2], or silicon-based lasers [3]. These SRSO structures may be successfully deposited by magnetron sputtering technique. After deposition, during annealing in a N_2_ atmosphere and 1,100°C temperature, the excess silicon in SRSO layer precipitates to form Si nanocrystals in nearly stoichiometric silicon dioxide matrix.

The structural quality of the matrix surrounding Si-NCs is very important since it influences the optical properties of Si-NCs [4]. For example, it has been shown that various defects present in the matrix may quench the emission originated from Si-NCs due to non-radiative recombination [5]. This is a serious problem from the point of view of applications, especially in the case of light-emitting devices. Besides the optical properties, due to differences in Si-NCs and SiO_2_ crystal structure, the matrix structural ordering may affect also the Si-NCs crystallinity and shape. It has been shown by first-principles calculations that the surrounding matrix always produces a strain on the nanocrystals, especially at the Si-NCs/SiO_2_ interface. According to theory, the amount of stress exerted on the nanocrystal is connected to the Si-NCs size [6] as well as to the number of oxygen per interface silicon [7]. These structural parameters can be controlled during deposition process by varying the excess silicon concentration in the SRSO matrix [8]. The structural properties of the Si-NCs may be then experimentally examined by means of the Raman spectroscopy, since the Si-Si bonding is Raman active. On the other hand, Si-O-Si bonds are active in the infrared (IR) region and therefore the matrix properties can be examined by means of the Fourier transform IR (FTIR) spectroscopy. In this work, we investigate the correlation between short-range structural order of the matrix and stress exerted on the Si-NCs by means of the Raman and FTIR spectroscopy. Our results indicate that there is a strong dependence of stress on the Si-NCs size and on the degree of short-range structural order of the matrix. We conclude that from the point of view of applications, a compromise has to be considered between good structural quality of the matrix and Si-NCs size.

## Methods

The SRSO films with a nominal thickness of 500 nm used for this study were deposited onto the quartz substrates by radio frequency reactive magnetron sputtering. The incorporation of Si excess was monitored through the variation of the hydrogen rate *r*_H_ = P_H2_ / (P_Ar_ + P_H2_). In this work we examined three samples deposited with *r*_H_ value equal to 10%, 30%, and 50%. The films were deposited without any intentional heating of the substrates and with a power density of 0.75 W/cm^2^. More details on the process can be found elsewhere [9]. All samples were subsequently annealed at 1,100°C for 1 h under N_2_ flux in order to favor the precipitation of Si excess and to induce Si-NCs formation.

The room temperature micro-Raman scattering was measured in vertical transmit, horizontal receive polarization, using single-stage spectrometer (T64000 Horiba Jobin Yvon, Longjumeau, France) equipped with a silicon charge-coupled device camera. An Ar^+^ laser (λ = 514.5 nm) was used as the excitation source. The lack of noticeable heating of the samples was assured by determination of the Stokes/anti-Stokes ratio. The FTIR spectra were collected using Nicolet iS10 spectrometer (Thermo Fisher Scientific Instruments, PA, USA). These measurements were conducted in attenuated total reflectance mode (ATR) using VariGATR accessory (Harrick Scientific Products Inc, NY, USA).

## Results and discussion

In our previous papers [9,10] we have reported results of structural investigations (including atomic force microscopy, X-ray diffraction, high-resolution electron microscopy or Rutherford backscattering) of SRSO films fabricated with the same technological parameters as the samples examined in the present study. The main conclusion of these investigations is that the deposition with *r*_H_ = 10% favors the formation of well-crystallized Si-NCs with average size of about 3 nm, whereas deposition with *r*_H_ = 50% favors formation of Si-NCs with size less than 2 nm. We have also shown that an increase of *r*_H_ results in a drop of the crystalline fraction of nanoclusters.

The samples examined in the present study were previously investigated by means of absorption spectroscopy [11]. The Tauc formula (α*E*) = *A* (*E* − *E*_g_)^*m*^ was used to estimate the optical band gap (*E*_g_) of these structures. The best fit to the experimental absorption data was obtained for *m* = 1/2, which corresponds to the directly allowed transition. It was found that the absorption edge is significantly blue-shifted from 3.76 eV for *r*_H_ = 10% to 4.21 eV for *r*_H_ = 50%, due to quantum confinement effect [12]. Moreover, it was found that below the optical band gap, the absorption spectra reveal long, exponentially decreasing absorption tails which can be described by Urbach equation: *α* = *C* exp(*E* / *E*_U_), where *E*_U_ is the characteristic Urbach energy. It was found that *E*_U_ increases as a function of *r*_H_ also increases from 73 meV (*r*_H_ = 10%) to 90 meV (*r*_H_ = 50%). For clarity, these results are summarized in Table 1.

**Table 1:** **The optical band gap (*E***_**g**_**) and Urbach energy (*E***_**U**_**) determined for the investigated samples**

***r***_**H**_**(%)**	***E***_**g**_**(eV) (*m*= 1/2)**	***E***_**u**_**(meV)**
10	3.75	73
30	3.97	75
50	4.22	90

**Figure 1:**
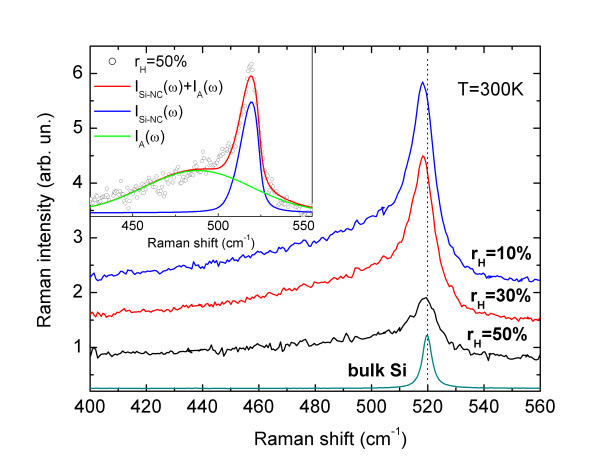
**Raman spectra measured for samples deposited with *r***_**H**_**equal to 10%, 30%, and 50%.** To compare, a reference spectrum of bulk Si is also shown. The spectra have been upshifted for clarity reasons. The inset shows fit of the phonon confinement model to the spectrum measured for *r*_H_ = 50% sample.

Figure 1 shows Raman spectra measured for samples deposited with *r*_H_ equal to 10%, 30%, and 50%. The spectra consist mainly of two bands: a broad low-frequency band (LF) with maximum at around 480 cm^−1^ and a narrower, asymmetrically broadened high-frequency (HF) peak centered between 518 and 519 cm^−1^. The LF band may be attributed to the amorphous silicon (a-Si) [13], whereas the HF originates from Si-NCs [14]. To compare we also show the reference spectrum of bulk Si with peak centered at *ω*_Si_ = 520 cm^−1^. Interestingly, despite the fact that the Si-NCs size decreases as a function of *r*_H_, the Raman line related to Si-NCs does not shift to the lower frequencies according to the predictions of the phonon confinement (PC) model [15]. Quite the contrary, it can be seen in Figure 1 that the Raman line slightly upshifts as a function of *r*_H_. In order to explore this rather surprising effect in more details, we have analyzed the HF Raman band using the PC model, following the approach proposed by Paillard et al. [16]:

(1) $$I_{\mathrm{Si}-\mathrm{NC}}\left(\omega \right)=C\int_{0}^{0.5}\frac{\sin^{2}\left[\left(\text{q}d/a_{0}\right)\pi \right]}{{\left[1-{\left(\text{q}d/a_{0}\right)}^{2}\right]}^{2}}\frac{d\text{q}}{{\left[\omega -\omega \left(\text{q}\right)\right]}^{2}+{\left(\Gamma_{0}/2\right)}^{2}},$$

where *d* is the Si-NC diameter, *a*_0_ = 0.543 nm is the Si lattice constant, q is the phonon wave vector expressed in 2π/*a*_0_ units and Г_0_ is the natural line width. As shown by Zi et al. [17], for small Si-NCs, the phonon confinement model can give a relatively good description of Raman frequency shifts, comparable to the predictions of the bond polarizability model. The high anisotropy of the phonon dispersion curves in silicon was also taken into account, using the averaged dispersion relation for the optical phonons, as proposed by Paillard et al.:

(2) $$\omega \left(\text{q}\right)=\sqrt{\omega_{c}^{2}-\frac{126,100\times {\text{q}}^{2}}{\text{q}+0.53}}$$

In the equation (2), the *ω*_c_ = *ω*_Si_ = 520 cm^−1^ is the optical phonon frequency at the Г point of the Brillouin zone of an unstressed bulk Si crystal. However, if stress is present in the material, the *ω*_c_ value changes [18]. Therefore, to retain all the information, during fitting procedure, we left *ω*_c_ as a free parameter together with *d*. Additionally, a Gaussian function was used to fit the LF band:

(3) $$I_{A}\left(\omega \right)=A_{A}\exp\left(-\frac{{\left[\omega -\omega_{A}\right]}^{2}}{2\delta_{A}^{2}}\right)$$

where *ω*_A_ is the LF band frequency, *A*_A_ denotes amplitude, and *δ*_A_ is related to Gaussian width. The overall model used to fit the Raman data is a sum of the amorphous and crystalline components:

(4) $$I\left(\omega \right)=I_{\mathrm{Si}-\mathrm{NC}}\left(\omega \right)+I_{A}\left(\omega \right).$$

Inset in Figure 1 shows an example of the fit obtained for *r*_H_ = 50% sample. It can be seen that the PC model accounts for the asymmetric shape of the Raman band of Si-NCs. This asymmetric shape is a result of a finite nanocrystals volume, which allows phonons away from the Brillouin zone center to contribute to the Raman scattering. Therefore, during the fitting procedure, we rely on two factors that directly depend on the Si-NCs size: the line-shape of the Raman band and the expected frequency of this band.

From the fit of Equation 4 to the Raman data, we obtained that the Si-NCs diameter *d* increases from about 2.4 nm for *r*_H_ = 50% to about 2.7 nm for *r*_H_ = 10% (the statistical error from the fitting procedure is less than 0.05 nm). The obtained results are in agreement with our expectations based on the structural data measured for similar samples. This result also confirms that the model given by Equation 1 can be used to estimate the Si-NCs size based on the Raman data.

The second important result obtained from the fit is *ω*_c_. For the unstressed Si crystal, this value equals to 520 cm^−1^. However, we obtained that *ω*_c_ increases as a function of *r*_H_ increases, from *ω*_c_ = 523 cm^−1^ for *r*_H_ = 10% to *ω*_c_ = 525 cm^−1^ for *r*_H_ = 50%. The obtained values strongly indicate that we deal with a compressive stress exerted on the Si-NCs which shifts the observed Raman lines towards higher wavenumbers [4]. Similar effect has been observed for Si-NCs obtained by chemical vapor deposition technique and annealed at 1,250°C [19]. Moreover, the observed rise of *ω*_c_ indicates that the stress increases as a function of *r*_H_. Assuming that the hydrostatic pressure of about 1 GPa results in approximately 1.88 cm^−1^ shift of the Raman line [20], we may estimate the maximum stress to be about 2.6 GPa for *r*_H_ = 50% sample. The obtained results also explain why we do not observe a clear downshift of the Raman frequency related to PC effect. Namely, the compressive stress increases as a function of *r*_H_ and compensates for the downshift due to the finite crystallite size. It is worth to note that PC effect has been actually observed for Si-NCs synthesized in the form of free-standing powder [21]. Therefore, the difficulties related to the observation of this effect in our case seem to be matrix-related. It should be also noted here that the obtained values of *ω*_c_ do not strongly depend on the PC model selection. To check this, we fitted the HF Raman band with another PC model proposed by Campbell et al. [15] (with a Gaussian weighting function instead of sinc). Although this model predicted overestimated Si-NCs sizes (4 nm for *r*_H_ = 50% and 5 nm for *r*_H_ = 10%), the obtained values of *ω*_c_ were similar (*ω*_c_ = 523 cm^−1^ for *r*_H_ = 10% and *ω*_c_ = 524 cm^−1^ for *r*_H_ = 50%). It should also be mentioned that both models are simplified since they do not take into account such effects as stress distribution or Si-NCs size distribution. Therefore, the estimated stress values should be treated as estimation.

In the next step, the Raman results were used to calculate the relative contribution of the HF (Si-NCs) and LF (a-Si) bands to the total Raman scattering, according to the following equations:

(5) $$\begin{array}{l} f_{\mathrm{HF}}=\frac{I_{\mathrm{Si}-\mathrm{NC}}}{I_{A}+I_{\mathrm{Si}-\mathrm{NC}}}\\ f_{\mathrm{LF}}=\frac{I_{A}}{I_{A}+I_{\mathrm{Si}-\mathrm{NC}}}, \end{array}$$

where the intensities *I*_Si-NC_ and *I*_A_ are defined as integrals over *ω* of Equations 1 and 3, respectively. We prefer to calculate the relative contributions instead of the absolute amorphous and crystalline fractions since, as shown by Ossadnik et al. [22], the Raman-based estimates of the latter can be very inaccurate.

**Figure 2:**
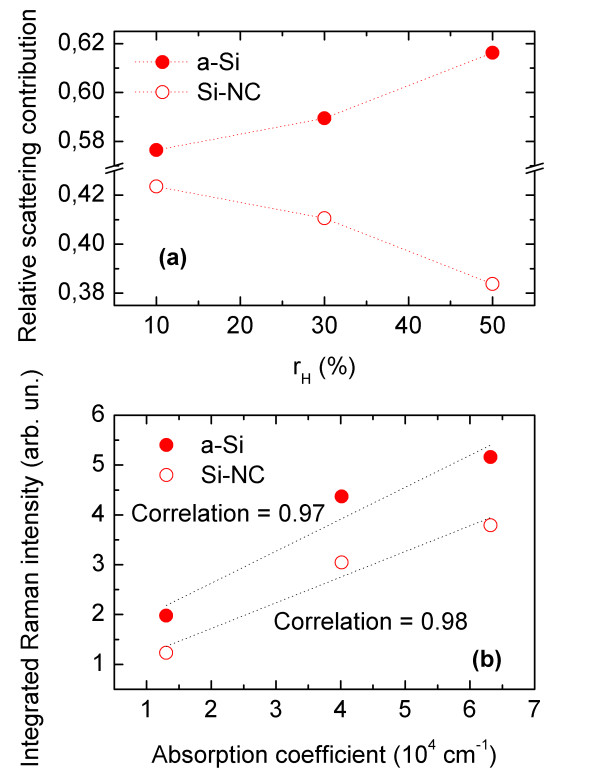
**Relative contribution of the HF (Si-NC) and LF (a-Si) Raman bands and their integrated Raman intensities.** (**a**) Relative contribution of the HF (Si-NC) and LF (a-Si) Raman bands to the total scattering intensity is shown as a function of *r*_H_. (**b**) Integrated Raman intensities of HF (Si-NC) and LF (a-Si) bands are shown as a function of absorption coefficient. Pearson’s correlation coefficients have been also shown for a-Si and Si-NC.

Figure 2a shows the relative contributions of the HF (Si-NCs) and LF (a-Si) bands to the total Raman scattering intensity as a function of *r*_H_. It can be seen that the relative contribution from Si-NCs drops with *r*_H_, which we believe reflects a relative drop of the crystalline fraction. Simultaneously, we observe a relative increase of the amorphous fraction with *r*_H_. These results are in agreement with our previous structural investigations for similar structures, where it has been shown that increase of *r*_H_ results in the increase of the amount of a-Si in the structures.

Figure 2b shows the integrated Raman intensities of Si-NCs and a-Si bands as a function of absorption coefficient (α). The absorption coefficient was determined at 4 eV (high-energy part of the absorption spectra). It can be seen that there is a linear correlation between α and the Raman intensity for Si-NCs as well as a-Si, with correlation coefficient equal to 0.98 and 0.97, respectively. Since both the Raman intensity and α depend linearly on the number of nanoparticles (e.g., Si-NCs), the obtained correlation indicates that the high-energy absorption is related to both: Si-NCs and a-Si. It should be also noted here that we obtained a strong correlation for the whole high-energy part of the absorption spectra (between 3 and 5 eV). Moreover, the correlation coefficient calculated for Si-NCs was always slightly higher than for a-Si. On the other hand, when energy drops approximately below 2.5 eV, the correlation coefficient also drops below 0.7. This result can be expected if we bear in mind that the estimated optical band gap exceeds 2.5 eV for all of the investigated structures (see Table 1). This result may also indicate that the low-energy part of the absorption spectra is (at least partially) related to some different structures e.g., defects in the matrix.

**Figure 3:**
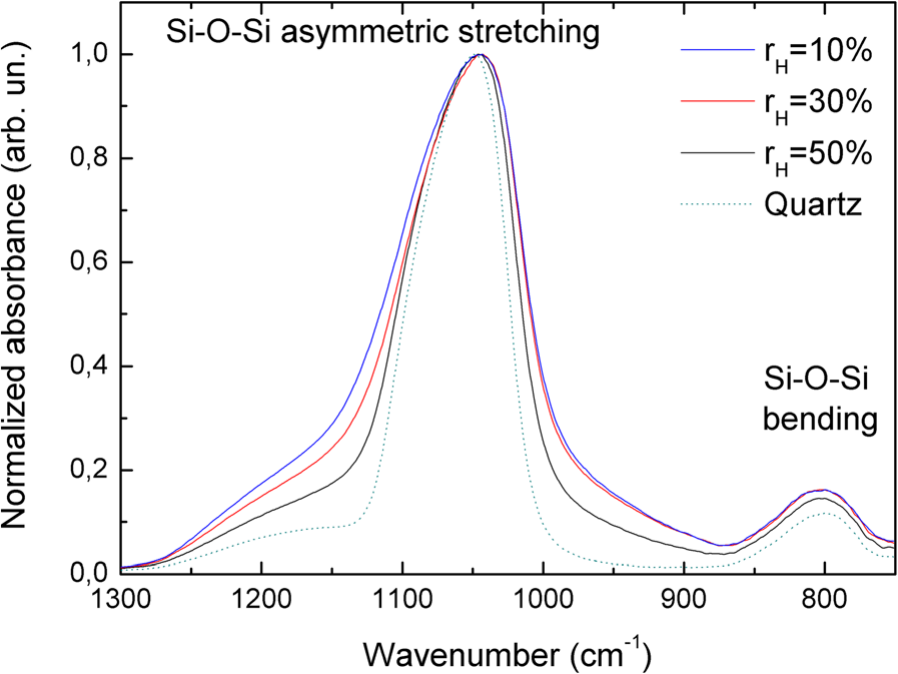
**Normalized FTIR spectra measured in ATR mode for samples deposited with different *r***_**H**_**.** The quartz reference spectrum is also shown for comparison (dotted line).

In order to explore the matrix properties for more details, we conducted FTIR measurements in ATR mode. Figure 3 shows normalized IR spectra obtained for the samples deposited with different *r*_H_. To compare, Figure 3 also contains a reference spectrum measured for pure quartz. In each case, the main band located in the range 1,000 to 1,300 cm^−1^ is associated with the asymmetric stretching Si-O-Si mode [23], where the bridging oxygen atoms move in the direction opposite to their Si neighbors and roughly parallel to the Si-Si lines. Moreover, the band around 800 cm^−1^ is identified as the bending Si-O-Si vibration [23] in which the oxygen move approximately at right angles to the Si-Si lines and in the Si-O-Si planes.

Figure 3 one can also see that the spectra of the samples deposited with excess silicon are much broader in comparison with the IR spectra of pure quartz. Moreover, the decrease of the *r*_H_ used during deposition leads to a significant broadening of the IR spectra. This effect can be related to the lowering of the degree of matrix structural order. It should be emphasized here that we consider a short-range order since the matrix is non-crystalline. A more quantitative definition of the matrix structural disorder is discussed below.

Recently, it has been well established that amorphous silica (a-SiO_2_) contains ring structures with different sizes [24]. The structure of a-SiO_2_ is a network of SiO_4_ tetrahedra containing irregular rings of order *n* < 6, where *n* is the number of Si atoms in a ring. In other words, the *n*-fold ring implies *n* Si atoms and *n* O atoms alternately connected in a loop. The irregularity of these rings is associated with the number of atoms in a loop (*n*-fold rings) as well as with the broad distribution of the Si-O-Si intertetrahedral bond angles *θ*[25]. In the framework of central-force network model, the distribution of *θ* can be ascribed entirely to the width of an IR or Raman mode [26]. This is because the mode angular frequency *ω*_i_ is related to *θ* by the following equation [26]:

(6) $$\omega_{i}\Delta \omega_{i}=\gamma_{i}\left(\alpha /2m_{X}\right)\sin\theta \Delta \theta$$

where Δ*ω*_i_ is the change of the *ω*_i_ mode angular frequency, Δθ is the variation of the angle *θ*, γ is a constant, α is a bond force constant and *m*_*x*_ denotes element mass. In this work we relate the structural disorder to a spread in *θ* and a wide distribution of *n* in the *n*-fold rings. This approach is clearly oversimplified since it does not account for the appearance of new modes induced by the disorder [27], which actually exist in an amorphous SiO_2_. Nevertheless, the above model enables us to understand the obtained results at least qualitatively and relate the observed broadening of the IR spectra to increase structural disorder of the matrix. This means that the siloxane rings structure is more diversified in the case of *r*_H_ = 10% samples, with various ring orders *n* and a large spread in the intertetrahedral angle *θ*.

We would like to note that there is a correlation between the structural order of the matrix and the magnitude of the compressive stress exerted on Si-NCs. Namely, the stress is higher when the structural order of the matrix increases. Although several explanations of the compressive stress exerted on Si-NCs in SRSO matrix have been proposed [19,28], we have not found any explanation which takes this effect into consideration. Here, we would like to suggest another possible origin of the compressive stress that accounts also for the observed correlation of the compressive stress magnitude on the structural order of the matrix.

Before we discuss this effect, we would like to note that after crystallization of a melted silicon nanoparticle, its volume increases by about 10% [29]. This is rather not typical behavior, related to the fact that silicon has greater density in the liquid state than in the solid state. Therefore, the phase-transition from liquid to crystalline state should lead to a compressive stress, when Si-NCs are embedded in a SiO_2_ matrix, despite the different thermal expansion coefficients of Si and SiO_2_. This also means that the compressive stress observed in our experiment may be indicative of the crystallization process, which proceeds through melting. This kind of process has been suggested by Hirasawa et al. [30] for Si nanoparticles synthesized by pulsed laser ablation, where the determined crystallization temperatures were in the range of 800 to 1,300 K (depending on the nanoparticle size). These temperatures are far below the melting point of bulk Si (1,683 K). In our case, the annealing temperature of 1,373 K is also well below the melting point of bulk Si and only slightly below the melting point of a-Si (1,420 K for relaxed a-Si [31]). However, it is well known that the melting temperature of a nanoparticle decreases significantly with size, as a consequence of the additional free energy contribution of the surface to the overall Gibbs free energy [32]. For example, it has been shown that free-standing Si nanoparticles with a size of 20 nm melt at around 1,000 K [32]. On the other hand, nanoparticles embedded in a matrix can exhibit both melting-point depression and enhancement [33], and the actual melting behavior depends on the nature of the interface between the nanoparticle and the matrix. It has been found that when the interface between the nanoparticle and the matrix is coherent, the thermal vibration of the surface (interface) atoms of the nanoparticle is suppressed. This suppression may prevent the melting of the nanocrystals’ surface and lead to an increase of the melting temperature. This kind of behavior has been found for lead nanocrystals in an aluminum matrix and was attributed to the lattice structures of the two crystals ‘locking up’, suppressing the vibration of the nanoparticles’ surface atoms [34]. Contrary to this, irregularly shaped and incoherent interfaces can be directly correlated with lowering of melting temperature of a nanoparticle [35].

In the investigated case, we expect that directly after deposition we deal with amorphous Si nanoparticles embedded in a disordered oxide matrix. Moreover, it is improbable that the sputtering technique allows deposit of coherent (epitaxial) interfaces between the amorphous nanoparticles and the matrix. Due to a large density gradient of the Si nanoparticles and the oxide host, when merged at their interface, the network topologies in either side deform in order to accommodate the transition [36]. Therefore, we expect the interfaces between Si nanoparticles and the matrix to be incoherent. This can be further supported by the latest findings of molecular dynamics simulations which have shown that the interface structure between Si-NCs and the matrix is generally highly porous on the silica side, making the contact with the Si-NCs discontinuous [37]. Taking this into account, we expect that the melting temperature of small, amorphous Si nanoparticles embedded in SRSO matrix might be depressed below the melting point of a-Si. If this is the case, melting of the nanoparticles may be possible at 1,100°C.

Having this in mind, we suggest the following origin of the compressive stress observed in our experiment. First of all, it should be emphasized that the matrix structure before and after annealing is not the same. Annealing at 1,100°C leads to phase separation on Si and SiO_2_ and the structural order of the matrix increases. Secondly, the crystallization of small a-Si nanoparticles takes place simultaneously to the matrix ordering. We suggest that for non-uniform structures obtained by sputtering, the crystallization may proceed through melting which in turn leads to volume expansion and compressive stress exerted on the Si-NC. Moreover, we may expect that the ability of Si-NCs to expand after crystallization should depend on the environment - particularly, on the degree of the structural order of the matrix (since expansion of the nanocrystal leads to matrix deformation). In other words, the matrix structure determines its ability to accommodate to the expanding Si-NCs. In this way, formation of a well-ordered matrix does not allow Si-NCs to expand freely, leading to a stronger compressive stress exerted on the Si-NCs. We deal with this situation for *r*_H_ = 50%, where the compressive stress is the strongest and the FTIR spectra are quite narrow, suggesting a higher structural order of the matrix than for the other samples. On the other hand, for larger Si-NCs (*r*_H_ = 10%), the structural order of the matrix is the lowest, resulting in a broad IR spectrum. This structural disorder indicates that the matrix can accommodate to the Si-NCs size/shape; therefore, compressive stress exerted on the Si-NCs is lowered.

Remarkably, the IR spectrum of pure quartz is much narrower than the spectra of the samples containing Si-NCs. It means that Si-NCs always introduce a large amount of the structural disorder to the matrix which may influence also the optical properties. This problem should be taken into account while designing structures for a particular application.

## Conclusions

In conclusion, we have shown that compressive stress is exerted on Si-NCs in SRSO samples deposited by radio frequency reactive magnetron sputtering. This stress may completely compensate for the phonon quantum confinement effects, resulting in the lack of a clear dependence of the Si-NCs-originated Raman line on the Si-NCs size. The compressive stress increases with the increasing *r*_H_ used during deposition. We relate the observed strong stress dependence on *r*_H_ to the changes of structural order of the matrix surrounding Si-NCs induced by *r*_H_ variation. The formation of an ordered matrix structure clearly competes with the formation of unstressed Si-NCs.

## Competing interests

The authors declare that they have no competing interests.

## Authors’ contributions

GZ, AP, and JM carried out the spectroscopic measurements as well as calculations. JC and FG designed and deposited the investigated samples. All authors read and approved the final manuscript.
